# Importance of Footwear for Preventing Xerosis and Hyperkeratosis in Older People with Psychiatric Disorders Living in an Institution

**DOI:** 10.3390/ijerph15040584

**Published:** 2018-03-24

**Authors:** Ana María Pérez-Pico, Félix Marcos-Tejedor, María José Iglesias-Sánchez, Raquel Mayordomo Acevedo

**Affiliations:** 1 Department of Nursing, Centro Universitario de Plasencia, Universidad de Extremadura, 10600 Plasencia, Cáceres, Spain; aperpic@unex.es (A.M.P.-P.); felixmt@unex.es (F.M.-T.); 2 Department of Pharmacology, Centro Universitario de Plasencia, Universidad de Extremadura, 10600 Plasencia, Cáceres, Spain; unexdedap@unex.es; 3 Department of Anatomy and Human Embryology, Centro Universitario de Plasencia, Universidad de Extremadura, 10600 Plasencia, Cáceres, Spain

**Keywords:** elderly care, carers/families, foot deformities, quality of care, shoes, psychiatric disorder

## Abstract

Few studies have focused on the relation between the use and characteristics of footwear and the presence of foot lesions in people with psychiatric disorders. This work analyzes the influence of different footwear habits on the presence of deformities and ungueal and dermal pathologies of the foot of institutionalized people with psychiatric disorders compared to people without these disorders. A transversal and observational study was conducted on 107 participants, divided into two groups who have used different types of shoes throughout their lives. The control group comprised 63 autonomous people who mainly use leather footwear and a study group of 44 institutionalized people with intellectual disabilities and psychiatric disorders who mainly use textile footwear. There were significant differences between populations. The group with psychiatric disorders presented more xerosis and hyperkeratosis. Footwear with inappropriate characteristics is a possible causal agent of skin alterations. Wearing footwear with quality textile uppers, e.g., fabric or felt, could influence the appearance of these alterations. Leather footwear is recommended for institutionalized people to reduce symptoms of xerosis and improve their quality of life.

## 1. Introduction

The influence of fashion on footwear has had a major role in the appearance of several foot disorders [1,2,3,4]. Some footwear characteristics have been linked to falls and alterations in the musculoskeletal system and the skin [5,6,7]. Health professionals have considered footwear characteristics as possible causal agents of foot disorders, as some injuries can be associated with ill-fitting footwear or a poor choice of shoes [7,8,9]. It is important to be aware that people with intellectual disabilities and psychiatric disorders have limited skills in communicating, caring for themselves, and performing activities of daily life as simple as ensuring their personal hygiene or choosing suitable footwear. They require care from health professionals (or guardians) who can remedy some of these shortfalls. Matsuba (2015) confirmed the importance of nursing care for people with disabilities living in institutions and/or at home [10]. The lack of adequate movement in these patients has also been associated with premature death [11]. 

Table 1 summarizes the characteristics we considered most appropriate from various works, to be taken into account when choosing shoes. The choice of footwear type normally depends on factors specific to each individual [3,16,17] and therefore when footwear does not meet these characteristics or is worn out, it is considered inappropriate. This table, as presented and used in this work, could help health professionals to determine whether the shoes of the people they treat have an influence on the foot disorders they present. 

Foot disorders can lead to more serious pathologies, particularly in older people, as a simple graze or chafing can become a serious lesion in people with systemic pathologies such as diabetes or circulatory disorders [17,18]. Professionals should also be able to inform these people (or the relatives responsible for them as legal guardians) about the importance of obtaining quality footwear with suitable characteristics to avoid foot pathologies.

## 2. Materials and Methods

### 2.1. Ethical Considerations

The study was conducted following the guidelines of the Declaration of Helsinki and was approved by the University Bioethics Committee (reference: 65/2012) and Health Centre (a residential care center which acted as the legal guardian of the participants, given that part of the study was performed there). Before the data was collected, informed consent was obtained from all participants.

### 2.2. Design and Sample

A transversal and observational study was conducted for two years (2014 to 2016). In total, 107 European Caucasian participants were analyzed. This is a level 3 study in accordance with the United States Agency for Health Care Research and Quality. This study evaluated primary subject's footwear and the subsequent pathologies they displayed. A cross-sectional, descriptive study was designed, with a sample that followed the inclusion criteria. 

The control population sample was recruited from people who attended the University Podiatric Clinic and met the following inclusion criteria: be autonomous; not have any intellectual disability or diagnosed chronic psychiatric disorder; not have been permanently hospitalized; and remember wearing the same type of footwear for the last 10 years. 

The study samples were recruited from all the people who regularly attend the podiatry service at the Health Centre. They met the following criteria: have a diagnosed intellectual disability (defined as mental retardation, the term used when there are limits to a person’s ability to learn at an expected level and function in daily life, starting in childhood or adolescence) according to the Diagnostic and Statistical Manual of Mental Disorders, Fifth Edition (DSM-5) from The American Psychiatric Association (APA) [19]. Using the criteria described in this manual, the following psychiatric disorders were considered: Psychotic Disorders (PsyD), Personality Disorders (PD), Disorders of Childhood and Adolescence (DCA), and Mood Disorders (MD). In all cases (intellectual disability and psychiatric disorder patients), the participants had been living in the residential center for more than 10 years. This allowed us to determine the type of footwear they usually wore in the last 10 years and for how many hours a day, as the Health Centre provides their shoes. 

A medical team from the Centre examined all patients so they could be characterized and diagnosed properly. An experienced podiatrist performed the examination of the foot disorders and footwear. All participants with diabetes at the start of the study had suffered from this condition for at least five years, but there were no significant differences in prevalence between populations (Table 2). None of the participants in the study presented peripheral neuropathy and none had renal disorders or foot ulcers. 

Exclusion criteria included for both populations: history of limb trauma, lesions, limitations of mobility, being immunocompromised, sleeping disorders, refusing to sign the consent form or being incapable of understanding the instructions necessary to carry out the study, and not meeting any of the criteria defined for inclusion. Table 2 shows the participant demographics of the study populations.

### 2.3. Procedure 

The same observer and podiatrist collected all data in both populations. To conduct the footwear study, we designed a table (Table 2) for use as an integral footwear evaluation tool. This table was based on the studies of Barton (2009), as they present good validity and reliability for critically evaluating footwear [4]. Studies by Menz (2000) were also taken into consideration [20].

The footwear characteristics were entered in Table 3 and when any of the variables analyzed was not met, the footwear was considered inappropriate. The shoes were classified into three types (collected in Table 1) according to how the participants used them: street footwear (walking shoes, oxford shoes, moccasin, boot, high/medium heel shoes, court shoes, or surgical/bespoke shoes), sports footwear (athletic shoes, runner shoes) and domestic use (home footwear or slipper). Each autonomous participant (control population) or patient caregiver (study sample) indicated which of the three types of footwear the participant used during most of the day (at least 8 h a day; in the case of slippers it was all day for up to 10 h) in the last 10 years. The podiatrist also assessed whether the usual footwear was suitable for the daily activity of each participant. To examine the fit of the footwear to the foot of the participant, a pedigraph was made and then cut out and inserted into the shoe to verify the fit. No differences were found between the measurements of the foot and the footwear participants used. 

The same podiatrist analyzed nail and skin pathologies and deformities and keratopathies in each participant of each population. The nail disorders identified were onychogryphosis, onychomycosis, parrot beak nails, onycholysis, ingrown toenails, nails with longitudinal lines, and hematomas. The toe deformities the participants presented were hallux valgus, claw toes, hammer toes, overlapping toes (infraductus or supraductus), and tailor’s bunions. The dermatopathies found were tinea pedis, moles, eczema, xerosis (X), verrucas, and cracking. The keratopathies observed were hyperkeratosis (Hk) and calluses in the dorsal, plantar, or interdigital zone.

### 2.4. Statistical Analysis

The data obtained were entered into a database for statistical analysis with the SPSS 19.0 (IBM, Madrid, Spain) for Windows software. The descriptive analysis of the dichotomic qualitative variables, with categories “Yes” and “No”, was based on the calculation of frequency tables or frequency distribution. The mean and standard deviation of the quantitative variables age and BMI were calculated and normality was checked using the Shapiro-Wilk test. As the distribution was not normal, the non-parametric tests for two independent samples were applied using the Mann-Whitney U test. The result showed that age is not a limiting factor in the study. To observe the relations between two categorical or qualitative variables, the Chi square independence test and Fisher’s exact test were used. For the statistical tests, a significance level of 5% was established. 

## 3. Results

The data obtained showed that the populations studied habitually wear different types of footwear. The control group normally wears street footwear, whereas the group living in an institution mainly uses home footwear (*p*-value < 0.001, Chi-square test) (Table 4).

By sex, significant differences between populations were obtained. The men in the control group use street footwear and it was noteworthy that no one in this group wears home footwear. However, in the group of men living in an institution, this type of footwear is used by more than one third of the population (*p*-value < 0.001, Chi-square test) (Table 4). 

A similar trend was observed with the women: those in the control group mainly use street footwear, compared to only 5.9% of the women living in an institution (*p*-value < 0.001, Chi-square test) (Table 4).

The podiatrist also assessed whether the footwear habitually worn was suitable for the daily activity. The results show that 74.6% of the control population and 86.4% of the institutionalized population used suitable shoes for the daily activity they do (*p*-value < 0.001). However, analysis of the specific characteristics of the footwear in accordance with Table 3 showed that the institutionalized population has a high percentage of footwear without appropriate characteristics (81.8% versus 14.3% of those used by the control population) (*p*-value < 0.001).

The material of the shoe upper was analyzed and interesting differences were observed between the populations. The control population uses footwear with leather uppers (*p*-value < 0.001, Chi-square test), whereas the population living in an institution most frequently wears shoes with textile uppers. By sex, there are differences between men and women of the population living in an institution, as the women prefer textile footwear and the men prefer leather. In the control population, both sexes preferred leather shoes (Table 5).

Worn out footwear appears to be more frequent among men in the population living in an institution, who have the highest percentage of shoes in a poor state of repair even though the shoe fitted the foot properly according to the pedigraph (Table 6).

When the different types of footwear and materials were related to the pathologies present in participants’ feet, a significant relation was observed between users of home footwear made of textile who had X and the presence of Hk in the dorsal area of the fourth toe. No relation was found for onychopathies or toe deformities (Table 7).

The prevalence of the digital deformities observed (described in materials and methods) does not differ between populations (data not shown) and no significant differences were shown when digital deformities were related to the type of shoe used by each participant (Table 7).

We analysed whether, in the population living in an institution, leather shoe uppers were less harmful than textile uppers. The data in Table 8 show that the institution population who used leather had less xerosis than those who used shoes made of textile or synthetic material. In patients living in an institution, the prevalence of xerosis was higher among those who wore footwear made from textile compared to footwear made from other materials. 

To analyze the possible influence of medication on the appearance of X and Hk, the population taking psychotropic medications was selected. Medicated participants who wore leather shoes presented a very low prevalence of X compared to participants who wore shoes with uppers of a different material (Table 9). In medicated participants who wore textile footwear, a high incidence of X was observed. This was the same for the entire population (Table 7). However, there were no significant differences for the presence of Hk in medicated patients in the dorsal zone of the fourth toe for any of the materials studied (Table 9), probably because of a biomechanical factor.

## 4. Discussion

The frequency of podiatry problems is higher in the elderly population in general, although this has been analyzed in few studies, specifically in a population with intellectual disabilities and/or psychiatric disorders [18,21,22]. Both sexes have foot problems, but the prevalence is higher among women. The causes of the presence of foot disorders include increased body mass, inappropriate footwear, lack of basic care, and overuse of the feet, which can have life-long consequences [2,4].

Burns (2002) demonstrated that older people admitted to a rehabilitation unit (72%) generally used ill-fitting footwear [23]. This agrees with our data, which show a high prevalence of footwear with inadequate characteristics in the group of institutionalized patients. The control group preferred street footwear and the group living in an institution preferred home footwear (Table 3).

The preference of the men living in an institution for street footwear could be because they are more autonomous and active than women, as occurs in the control group of this work. In the case of women, it must be taken into account that they seldom leave the center and are highly sedentary. Moreover, the people responsible for choosing their footwear prioritize comfort over aesthetics, whereas the women in the control group choose their own shoes and put aesthetics first (personal observation). This concurs in several studies in which women aged 60 to 80 years were found to put aesthetics before comfort when choosing footwear [3,24,25].

Our results agree with those of Menz (2000), who reported that people who are more active wear street footwear, and Alcántara (1998), who showed that sedentary people and the elderly use home footwear more frequently [14,20].

Some studies found a relation between narrow footwear and certain pathologies and deformities in the forefoot, as well as foot pain [1,2,4], although from the results obtained in this study it cannot be assumed that footwear is an influencing factor in the appearance of toe deformities in general for the populations studied.

We found no relation between the type of footwear used and any of the nail disorders studied, but we did find a relation between the presence of Hk in the dorsal zone of the toes and X in people who use home footwear with textile uppers. The model of home footwear they use probably has a rounded, slightly narrower toe box, and as the fabric is not elastic, it can rub the dorsal zone of the toes when they are in dorsal flexion during toe-off and produce the alteration [2,14,18]. Branthwaite (2013), in a study on footwear, concluded that both the shape of the toes and the toe box are involved in the appearance of Hk and increased pressure in the dorsal zone of the toes [13]. This author stressed the importance of seeking good advice when choosing footwear, concurring with the data shown in this study in the relation found with Hk in the dorsal zone of the fourth toe (Table 6). The participants most affected by X and hyperkeratosis Hk are those who live in an institution. Some authors argue that this population presents very different characteristics from the control group, such as a lack of autonomy because of their disability (in most cases) and multi-medication with psychotropic drugs [26,27]. However, in this study, we can compare the populations because they have similar daily activity according to the Barthel scale (Table 2), are of the same race, and have a similar BMI, age, and comorbidities. Most footwear used by people in the institutionalized group is textile and is not elastic but absorbent, and can also cause chafing and dry skin. This explains the direct relation between this type of footwear and Hk and X. The data comparing the material of the footwear among patients living in an institution support the idea that textile is directly associated with the prevalence of X in these patients (Table 8). 

Berger (2013) reported that 50% of older patients have X leading to pruritus and generalized scaling, which can cause lesions from scratching and the loss of skin integrity and protection [28]. All of this can result in a risk of serious health problems in some patients, such as skin ulcers or lower limb amputation. Other studies reported the higher prevalence of X in older people living in rest homes and stressed the importance of treating it to prevent ulcers and stasis dermatitis [29,30]. Our data show no relation between these pathologies and age as they are comparable (Table 2) and all the participants have a mean age above 62 years. However, they do indicate that at these ages, the use of inappropriate footwear or footwear without leather uppers favors the presence of X and Hk.

Home footwear with textile uppers does not meet two of the original characteristics for which humans devised footwear [2,24], i.e., protecting feet from blows and severe weather. Direct microtraumas can cause lesions to the nail apparatus or the foot in the long term. Textile footwear lets air circulate more freely, which can favor dry skin and the appearance of X. However, street footwear incorporates stiffeners and materials that help to protect the feet from injury and the weather. Leather is more breathable than textile and adapts naturally to the foot.

Some studies have demonstrated the influence of medication with psychotropic drugs on foot disorders [31,32,33]. Because medication could have influenced the results obtained, we analyzed the participants who took these drugs, statistically demonstrating that the type of footwear was a factor influencing the prevalence of xerosis. Therefore, medication and footwear could be considered related influencing factors in the development of this pathology.

This study presents new findings that agree with some authors who have related textile shoe uppers to the appearance of skin lesions. A recent work [18] on patients with Alzheimer’s Disease showed that ill-fitting footwear (home wear) influences the presence of foot problems. Another article [34] addressing this reported that the ergonomic properties of protective footwear worn by members of certain professions at work could be enhanced by using appropriate textile components. This suggests that there are inappropriate textile components that may be harmful. Our results could have the limitation of not demonstrating whether other textile materials can prevent the appearance of these skin pathologies. This could be examined in further studies and would help the dependent population in particular to acquire better footwear. Domestic leather shoes do exist and these could be the best option for institutionalized elderly persons who suffer from xerosis and hyperkeratoses, which is what we have concluded in light of the data shown in this work.

## 5. Conclusions

Footwear with inappropriate characteristics is a possible causal agent of skin pathologies such as X and Hk. Wearing footwear with quality textile uppers, e.g., fabric or felt, could influence the appearance of these pathologies. We therefore consider it important to wear shoes made from high quality, natural materials such as leather or suede. Health professionals should take into account the results obtained in this work to recommend the best footwear to patients in their care, especially dependent patients such as those suffering from psychiatric disorders and who are also medicated. This can help to improve the quality of life of dependent and institutionalized patients.

## Figures and Tables

**Table 1:** Appropriate characteristics for street footwear, home footwear, and sports shoes.

Components of a Footwear	Street Footwear	Home Footwear	Sports Shoes
Toe box	Closed, wide, adapted to the foot, room for the toes, not pointed [12,13].	Closed, fairly stiff, adapted to deformities [12,13].	Closed, round, wide, adapted to the foot, room for toes [12,14].
Heel height	Men 2–2.5 cm. Women 2–3 cm [12].	Low and wide. Men and women 2 cm [12,13].	No heel or added insoles [15].
Material of uppers	Quality, natural materials: leather, suede [12,14].	Washable, synthetic, flexible, light, warm for winter and breathable for summer [12,13,14].	Elastic (normally leather) and breathable [14,15].
Sole	Materials: natural or manmade rubber, polyurethane. Sole thickness: 8–10 mm. Non-slip features: tread for better grip on the floor [12,14].	Flexible but stable. Cushioning. Materials: microcellular rubber or polyurethane, natural or manmade rubber. Non-slip features: tread to aid gripping [12,14].	Light, cushioning, with non-slip features: tread for better grip on the ground [12,14,15].
Width and length	Appropriate volume for the foot and metatarsal-phalangeal zone. Length: 10–20 mm men’s footwear and 10–15 mm women’s footwear [5,12].	Suitable for deformities and the volume of the foot. Length: 10–20 mm men’s footwear and 10–15 mm women’s footwear [12,14].	Suitable for the volume of the foot [12,14].
Seams	No harmful seams [12,14,16].	No harmful seams [12,14,16].	No harmful seams [12,14,16].
Fastenings	High, with laces or Velcro [12,14].	Velcro or elastic [12,14].	Laces or Velcro, with tongue [2,12].
Quarter	Stiff heel counter, closed [12,14].	Light heel counter without reinforcing, closed [12,14].	Heel counter, closed [12,14].

**Table 2:** Comparison of participant demographics.

Participant Demographics				
Analyzed Variables	Categories	CP	IP	*p*-Value
Sex	Mens	44.4%	61.4%	0.116
	Women	55.6%	38.6%	
Age (years)	Mens	64.75 ± 12.036	63.70 ± 9.953	0.561
	Women	60.06 ±13.911	72.35 ± 9.360	0.008 *
	Total	62.14 ±13.219	67.05 ± 10.519	0.141
BMI	Severely underweight (<16)	0.0%	0.0%	0.207
	Moderately underweight (16–16.99)	0.0%	0.0%	
	Acceptably underweight (17–18.49)	0.0%	0.0%	
	Normal (healthy weight) (18.5–24.99)	35.5%	22.7%	
	Overweight (25–29.99)	46.8%	50.0%	
	Obese Class I (30–34.99)	16.1%	18.2%	
	Obese Class II (35–40)	1.6%	9.1%	
	Obese Class III (>40)	0.0%	0.0%	
Diabetes mellitus	Total	11.1%	20.5%	0.270
Hypertension	Total	28.6%	13.6%	0.099
Hypercholesterinemia	Total	30.2%	25.0%	0.663
Type of foot (footprints)	Cavus foot	28.1%	40.0%	0.418
	Flat Foot	6.3%	4.0%	
	Normal Foot	43.8%	24.0%	
	Asymmetry in Feet	21.9%	32.0%	
Dx psychiatric disorder	None	88.9%	0.0%	0.000 *
	PsyD	0.0%	25.0%	
	PD	0.0%	29.5%	
	DCA	0.0%	29.5%	
	MD	11.1%	15.9%	
Barthel Index (BI)	Total dependency (<20)	0.0%	0.0%	0.098
	Severe dependency (21–60)	0.0%	0.0%	
	Moderate dependency (61–90)	1.6%	4.5%	
	Slight dependency (91–99)	7.9%	20.5%	
	Fully Independent (100)	90.5%	75.0%	

Note: % = Percentages, kg = Kilograms, CP = Control Population, IP = Institutionalized Population, RF = Right Foot, LF = Left Foot, Dx = Diagnosis, PsyD = Personality disorders, DCA = Disorder of childhood and adolescence, MD = Mood Disorders, * = Statistically significant.

**Table 3:** Table for entering the characteristics of footwear worn by study participants.

Characteristics of Street Footwear		
YES	Shoe upper: natural, quality materials	NO
YES	Volume: appropriate for the foot	NO
YES	Heel: appropriate height	NO
YES	Sole: light with multidirectional tread, neither too flexible nor too stiff	NO
YES	Seams: not harmful	NO
YES	Quarter: closed, with heel counter	NO
YES	Toe box: closed, not pointed	NO
YES	In good state of repair	NO
	Characteristics of Home Footwear	
YES	Shoe upper: flexible materials	NO
YES	Volume: appropriate for the foot	NO
YES	Heel: appropriate height	NO
YES	Sole: Microcellular rubber, natural or manmade rubber, non-slip	NO
YES	Seams: not harmful	NO
YES	Quarter: closed, with heel counter	NO
YES	Toe box: closed, not pointed	NO
YES	In good state of repair	NO
	Characteristics of Sports Shoes	
YES	Shoe upper: flexible, breathable materials	NO
YES	Volume: appropriate for the foot	NO
YES	Heel: none	NO
YES	Sole: cushioning and non-slip	NO
YES	Seams: not harmful	NO
YES	Quarter: closed, with heel counter	NO
YES	Shoe box: closed, not pointed, room for toes	NO
YES	In good state of repair	NO

**Table 4:** Footwear type percentages by population and sex.

Population/Sex	N	Street	N	Sports	N	Home	Chi-Square
CP	57	90.5%	5	7.9%	1	1.6%	<0.001 *
PI	14	31.8%	5	11.4%	25	56.8%	
	**N**	**Street**	**N**	**Sports**	**N**	**Home**	**Chi-Square**
Men CP	26	92.9%	2	7.1%	0	0%	<0.001 *
Men PI	13	48.1%	4	14.8%	10	37%	
	**N**	**Street**	**N**	**Sports**	**N**	**Home**	**Chi-Square**
Women CP	31	88.5%	3	8.6%	1	2.9%	<0.001 *
Women PI	1	5.9%	1	5.9%	15	82.2%	

Note: * = statistically significant; CP = Control Population; PI = Population living in an institution; % = Percentage; N = Number.

**Table 5:** Shoe upper material by sex, age and study population.

Type of Material	N	% CP	N	% PI	Chi-Square
Leather	56	77.8%	16	22.2%	<0.001 *
Textile	1	3.7%	26	96.3%	
Synthetic or other	6	75%	2	25%	
**PI**	**N**	**% Men**	**N**	**% Women**	**Chi-Square**
Leather	15	93.8%	1	6.3%	0.004 *
Textile	11	42.3%	15	57.7%	
Synthetic or other	1	50%	1	50%	
**CP**	**N**	**% Men**	**N**	**% Women**	**Chi-Square**
Leather	25	48.2%	29	51.8%	0.223
Textile	0	0%	1	100%	
Synthetic or other	1	16.7%	5	83.3%	

* = Statistically significant; CP = Control Population; PI = Population living in an institution; % = Percentage; N = Number.

**Table 6:** State of repair of shoes worn, by sex and study population.

Footwear State of Repair	Poor	%	Good	%	Fisher Exact Test
CP	1	1.6%	62	98.4%	0.008 *
PI	7	15.9%	37	84.1%	
**Men**	**Poor**	**%**	**Good**	**%**	**Fisher Exact Test**
CP	0	0%	28	100%	0.004 *
PI	7	25.9%	20	74.1%	
**Women**	**Poor**	**%**	**Good**	**%**	**Fisher Exact Test**
CP	1	2.9%	34	97.1%	0.673
PI	0	0%	17	100%	

* = Statistically significant; CP = Control Population; PI = Population living in an institution; % = Percentage; N = Number.

**Table 7:** Upper part of the table: Relation between toe deformities, alterations of the skin or skin appendages, and footwear types of all study participants and pathologies associated with their use. Lower part: Relation between toe deformities, alterations of the skin or skin appendages, and shoe upper materials for all study participants and pathologies associated with their use.

Footwear Type and Foot Pathologies					
	N	% Toe deformity	N	% No Toe Deformity	Chi-Square
Street footwear	54	76.1%	17	23.9%	0.426
Home footwear	18	69.2%	8	30.8%	
Sports shoes	9	90%	1	10%	
	**N**	**% Keropathies**	**N**	**% No keropathies**	**Chi-Square**
Street footwear	62	87.3%	9	12.7%	0.662
Home footwear	21	80.3%	5	19.2%	
Sports shoes	9	90%	1	10%	
	**N**	**% Dermatopathies**	**N**	**% No dermatopathies**	**Chi-Square**
Street footwear	35	49.3%	36	50.7%	0.107
Home footwear	19	73.1%	7	26.9%	
Sports shoes	5	50%	5	50%	
	**N**	**% Onychopathies**	**N**	**% No onychopathies**	**Chi-Square**
Street footwear	34	47.9%	37	52.1%	0.171
Home footwear	18	69.2%	8	30.8%	
Sports shoes	5	50%	5	50%	
	**N**	**% Xerosis**	**N**	**% No xerosis**	**Chi-Square**
Street footwear	13	18.3%	58	81.7%	0.000 *
Home footwear	17	65.4%	9	34.6%	
Sports shoes	3	30%	7	70%	
	**N**	**% Dorsal Hk 4th toe**	**N**	**% No dorsal Hk 4th toe**	**Chi-Square**
Street footwear	0	0%	71	100%	0.008 *
Home footwear	3	11.5%	23	88.5%	
Sports shoes	0	0%	10	100%	
**Shoe Upper Material and Foot Pathologies**					
	**N**	**% Toe deformity**	**N**	**% No toe deformity**	**Chi-Square**
Leather	54	75%	18	25%	0.233
Textile	19	70.4%	8	29.6%	
Synthetic or other	8	100%	0	0%	
	**N**	**% Keropathies**	**N**	**% No keropathies**	**Chi-Square**
Leather	62	86.1%	10	13.9%	0.415
Textile	22	81.5%	5	18.5%	
Synthetic or other	8	100%	0	0%	
	**N**	**% Dermatopathies**	**N**	**% No dermatopathies**	**Chi square**
Leather	34	47.2	38	52.8	0.060
Textile	19	70.4	8	29.6	
Synthetic or other	6	75	2	25	
	**N**	**% Onychopathies**	**N**	**% No onychopathies**	**Chi-Square**
Leather	33	45.8%	39	54.2%	0.080
Textile	19	70.4%	8	29.6%	
Synthetic or other	5	62.5%	3	37.5%	
	**N**	**% Xerosis**	**N**	**% No Xerosis**	**Chi-Square**
Leather	13	18.1%	59	81.9%	0.000 *
Textile	17	63%	10	37%	
Synthetic or other	3	37.5%	5	62.5%	
	**N**	**% Dorsal Hk 4th toe**	**N**	**% No dorsal Hk 4th toe**	**Chi-Square**
Leather	0	0%	72	100%	0.010 *
Textile	3	11.1%	24	88.9%	
Synthetic or other	0	0%	8	100%	

* = Statistically significant; % = Percentage; N = Number; Hk = Hyperkeratosis.

**Table 8:** Relation between alterations of the skin and shoe upper material in institutionalized participants with intellectual disability and psychiatric disorder.

People with Intellectual Disability and Psychiatric Disorder			
	% Xerosis	% No xerosis	Fisher exact test
Leather footwear	25.0%	75.0%	0.031 *
Non-leather footwear	60.7%	39.3%	
	**% Dorsal Hk 4th toe**	**% No dorsal Hk 4th toe**	**Fisher exact test**
Leather footwear	0.0%	100%	0.290
Non-leather footwear	10.7%	89.3%	
	**% Xerosis**	**% No xerosis**	**Fisher exact test**
Textile footwear	61.5%	38.5%	0.036 *
Non-textile footwear	27.8%	72.2%	
	**% Dorsal Hk 4th toe**	**% No dorsal Hk 4th toe**	**Fisher exact test**
Textile footwear	11.5%	88.5%	0.258
Non-textile footwear	0%	100%	
	**% Xerosis**	**% No xerosis**	**Fisher exact test**
Synthetic footwear	50.0%	50.0%	1.000
Non-synthetic footwear	47.6%	52.4%	
	**% Dorsal Hk 4th toe**	**% No dorsal Hk 4th toe**	**Fisher exact test**
Synthetic footwear	0%	100%	1.000
Non-synthetic footwear	7.1%	92.9%	

* = Statistically significant; % = Percentage; N = Number; Hk = Hyperkeratosis.

**Table 9:** Relation between shoe upper material and X and Hk in the dorsal zone of the fourth toe (4th) in people taking psychotropic medication from both populations.

People Populations Taking Psychotropic Medication			
	% Xerosis	% No xerosis	Fisher exact test
Leather footwear	23.8%	76.2%	0.037 *
Non-leather footwear	57.7%	42.3%	
	**% Dorsal Hk 4th toe**	**% No dorsal Hk 4th toe**	**Fisher exact test**
Leather footwear	0%	100%	0.242
Non-leather footwear	11.5%	88.5%	
	**% Xerosis**	**% No Xerosis**	**Fisher exact test**
Textile footwear	58.3%	41.7%	0.039 *
Non-textile footwear	26.1%	73.9%	
	**% Dorsal Hk 4th toe**	**% No dorsal Hk 4th toe**	**Fisher exact test**
Textile footwear	12.5%	87.5%	0.234
Non-textile footwear	0.0%	100%	
	**% Xerosis**	**% No Xerosis**	**Fisher exact test**
Synthetic footwear	50.0%	50.0%	1.000
Non-synthetic footwear	42.2%	57.8%	
	**% Dorsal Hk 4th toe**	**% No dorsal Hk 4th toe**	**Fisher exact test**
Synthetic footwear	0.0%	100%	1.000
Non-synthetic footwear	6.7%	93.3%	

* = statistically significant; % = Percentage; N = Number; Hk = Hyperkeratosis.
